# Impact of Continuous Positive Airway Pressure on Patient Outcomes in Acute Cardiogenic Pulmonary Edema Within Physician-Led Prehospital Care

**DOI:** 10.3390/medsci13010005

**Published:** 2025-01-01

**Authors:** Tatjana Jevtić Drkić, Armin Šljivo, Kenan Ljuhar, Amela Ahmić Tuco, Lamija Hukić Fetahović, Emina Karamehić, Amna Palikuća Ljuhar, Jasna Husejinbegović Musić, Šejla Brković Jusufbegović, Edin Jusufbegović, Selma Terzić Salihbašić, Melica Imamović Bošnjak, Riada Blažević, Amina Valjevac

**Affiliations:** 1 Institute for Emergency Medical Assistance of Canton Sarajevo, 71000 Sarajevo, Bosnia and Herzegovina; sljivo95@windowslive.com (A.Š.); kenan.ljuhar@gmail.com (K.L.); amna.palikuca@gmail.com (A.P.L.); jasnamusic6@gmail.com (J.H.M.); sejlapanikyok@gmail.com (Š.B.J.); edin.wi@gmail.com (E.J.); selmaterzic96@gmail.com (S.T.S.);; 2 Clinical Center of University of Sarajevo, 71000 Sarajevo, Bosnia and Herzegovina; amelatuco@gmail.com (A.A.T.); lamija.hukic@hotmail.com (L.H.F.); 3 Public Institution Department for Healthcare of Women and Maternity of Sarajevo Canton, 71000 Sarajevo, Bosnia and Herzegovina; karamehic.emina@gmail.com; 4 Cantonal Hospital Zenica, 72000 Zenica, Bosnia and Herzegovina; imamovic.melica@icloud.com; 5 Faculty of Medicine, University of Sarajevo, 71000 Sarajevo, Bosnia and Herzegovina; amina.valjevac@mf.unsa.ba

**Keywords:** CPAP, cardiogenic pulmonary edema, prehospital care, emergency medicine, clinical outcomes

## Abstract

Background: CPAP has been shown to be particularly beneficial in the management of acute cardiogenic pulmonary edema by reducing both preload and afterload, thus decreasing the work of breathing and improving oxygenation. Methods: This study was a prospective observational study, conducted in the period from 2022 to 2024, assessing the effectiveness and safety of prehospital CPAP therapy use in patients with acute cardiogenic pulmonary edema, administered alongside standard care. Results: In this study, 50 patients with acute cardiogenic pulmonary edema were treated by physician-led emergency teams in the Canton of Sarajevo. CPAP significantly improved clinical parameters across all time points. Systolic blood pressure decreased from 151.0 ± 41.0 mmHg at initial contact to 138.4 ± 32.0 mmHg before transportation and further to 130.2 ± 28.5 mmHg upon hospital admission (*p* < 0.001). Diastolic pressure dropped from 85.6 ± 17.2 mmHg to 81.1 ± 15.2 mmHg before transportation (*p* = 0.018), with a slight further decrease to 80.2 ± 13.9 mmHg (*p* = 0.083). Heart rate fell from 114 ± 26.4 bpm to 111.3 ± 24.9 bpm before transportation (*p* = 0.003) and finally to 99.5 ± 18.2 bpm before hospital admission (*p* < 0.001). Respiratory rate decreased from 31.0 ± 10.2 to 28.0 ± 10.5 breaths/min (*p* = 0.002) and further to 22.6 ± 7.3 breaths/min (*p* < 0.001). End-tidal CO_2_ levels increased from 28.0 mmHg (23.5; 33.5) to 30.0 mmHg before transportation (*p* < 0.001), and to 35.0 mmHg (32.0; 37.5) before hospital admission (*p* < 0.001). Oxygen saturation improved from 79.0% (72.0; 81.0) to 84.0% before transportation (*p* < 0.001) and reached 94.0% (91.0; 98.2) before hospital admission (*p* < 0.001). VAS scores for dyspnea significantly dropped from 8.0 (6.0; 8.2) at initial contact to 6.0 (4.0; 8.0) before transportation (*p* < 0.001) and further to 4.0 (3.0; 5.0) before hospital admission (*p* < 0.001), indicating substantial symptom relief. ECG findings remained stable throughout the intervention. Conclusions: Prehospital CPAP therapy significantly improved clinical outcomes in cardiogenic pulmonary edema, including reductions in blood pressure, heart rate, respiratory rate, and enhanced oxygenation and symptom relief. These findings support its broader use in emergency care, even during short transport times.

## 1. Introduction

Sudden dyspnea is a common clinical presentation that frequently necessitates the activation of emergency medical services, with “difficulty breathing” identified as one of the most prevalent chief complaints reported to dispatch [1].

Acute pulmonary edema, often resulting from underlying conditions, such as congestive heart failure, myocardial infarction, hypertensive crisis, arrhythmias, pulmonary thromboembolism, infection, and cardiac tamponade, is a significant cause of non-traumatic breathing difficulty [2]. This condition is typically managed with high-flow oxygen, standard pharmacological interventions, and urinary catheterization for diuresis monitoring (in accordance with the 2021 ESC guidelines for the diagnostics and treatment of acute and chronic heart failure) [3]; however, patients often deteriorate rapidly during prehospital transport, prompting the growing use of continuous positive airway pressure (CPAP) therapy in the field [4]. CPAP is administered to conscious patients who are capable of cooperating and are spontaneously breathing through a noninvasive facemask, with a positive airway pressure applied throughout the entire respiratory cycle [5,6]. CPAP provides several advantages for patients with pulmonary edema, particularly acute cardiogenic pulmonary edema. It enhances oxygenation by increasing end-expiratory lung volume and recruiting collapsed alveoli, thereby improving gas exchange [7]. Additionally, CPAP reduces the work of breathing by supplying positive pressure, which alleviates respiratory distress and fatigue [8].

CPAP also facilitates fluid reabsorption in the pulmonary interstitium, diminishing excess fluid in the lungs [8]. It improves lung mechanics by maintaining airway patency and enhancing lung compliance. Furthermore, CPAP decreases preload by reducing venous return to the heart, alleviating symptoms of heart failure and pulmonary congestion [9].

However, the benefits of the early initiation of CPAP in prehospital settings in Bosnia and Herzegovina have not yet been recognized. Currently, the organization, equipment, and education of the staff of emergency medical services (EMSs) differ widely across the country and therefore different levels of life support are provided. Furthermore, this means that the prehospital field treatment of acute cardiogenic pulmonary edema varies greatly from region to region. All EMS teams at the Institute for Emergency Medical Assistance of Canton Sarajevo are physician-staffed and fully equipped, therefore allowing for much more extensive treatment options than in many regions of Bosnia and Herzegovina.

In many Western countries, the National Association of EMS Physicians recommends initiating noninvasive ventilation, such as CPAP therapy, in prehospital settings [10]. Studies [7,8,9,10] on prehospital CPAP therapy mainly originate from these countries, but a majority of them report lower rates of intubation and lower mortality despite the fact that ambulances are not physician-staffed and the indication for administering CPAP is made by either a paramedic or an emergency medical technician.

In comparison, studies on the prehospital application of CPAP and its efficacy and safety have not yet been carried out in Bosnia and Herzegovina. This research intends to reflect our experience with prehospital CPAP therapy in a physician-led emergency service. Therefore, the findings of this study could pave the way for the more widespread use of noninvasive ventilation in Bosnia and Herzegovina. The aim of this study is to evaluate the impact and safety of the prehospital administration of CPAP as an additional measure to standard care.

## 2. Materials and Methods

This study was a prospective observational study, conducted in the period from May 2022 to November 2024, assessing the effectiveness, adherence, and safety of prehospital CPAP therapy in patients with acute cardiogenic pulmonary edema, administered alongside standard care. The primary objective was to assess the effectiveness of CPAP therapy, which was carried out by monitoring the changes in peripheral oxygen saturation (SpO_2_) and the respiratory rate. The secondary objective was to evaluate the adherence and safety of prehospital CPAP treatment, which was carried out by observing the incidence of the discontinuation of CPAP treatment and the adverse effects thereof. This study was conducted in accordance with the Helsinki Declaration and was approved by the Ethics Committee of the Institute for Emergency Medical Assistance of Canton Sarajevo on 18 May 2022, under approval number 3085, and confirmed and extended on 3 July 2024, under approval number 4467/24-2. The training of physician-led teams regarding guidelines, indications for use, and the technical aspects of CPAP mask therapy application as well as data gathering and documentation was carried out.

### 2.1. Study Setting

The regional EMS, which is serviced by a single public agency—the Institute for Emergency Medical Assistance of Canton Sarajevo—covers an urban area of 1276.9 km^2^ and (according to the 2013 consensus) 413,593 inhabitants. The organization is structured as a single-tier system, each ambulance being staffed by a physician. On a 24 h basis, there are 10 physician-staffed advanced life support care ambulance units, strategically placed at earlier established geographical points in order to reach patients sooner. During this 24 h time frame, roughly 100–120 interventions are carried out, with the attending physician deciding on treatment and the necessity of transport to a secondary or tertiary care facility. The call center (dispatch center) which accepts patient calls is operated by an emergency medical specialist and two emergency medical technicians with over 10 years of field experience, using the Australasian triage scale. All interventions are recorded into the official ambulance protocols (one in dispatch and another in the ambulance car), with the patients’ anamnesis, vital signs, circulatory status, respiratory status, EKG, physical findings, treatment administered, transport times, and changes during transport being recorded in the ambulance protocol.

### 2.2. Patients and Standard Therapy Protocol

The inclusion criteria were as follows: (i) patients over the age of 18 years old, (ii) conscious, (iii) able to cooperate, and (iv) patients assessed by a physician as suffering from acute cardiogenic pulmonary edema based on physical examination findings and the presence of the following symptoms and signs—acute onset dispnea or tachipnea (respiratory rate > 25/min), orthopnea, fine crackles (rales) heard upon auscultation usually starting at lung bases, increased work of breathing, use of accessory muscles or abdomen paradox, possible hemoptysis or rust-colored sputum, tachycardia, desaturation (SpO_2_ < 94%), pallor or mottled skin, leg or abdominal swelling, marked diaphoresis, and accompanying anxiety with a sense of suffocation. The exclusion criteria were: (i) age under 18 years, (ii) a lowered level of consciousness leading to the inability to cooperate, disorientation, nausea, vomiting, trauma to the face, any condition requiring endotracheal intubation (respiratory or circulatory arrest), (iii) suspicion of pneumothorax, hypotension (systolic blood pressure under <90 mmHg), suspicion of a cerebrovascular insult, foreign body airway obstruction, massive hemorrhage, and epiglottitis. The criteria for the termination of CPAP therapy were: insufficient increase in peripheral oxygen saturation, respiratory exhaustion, impending circulatory collapse, and increased agitation or disorientation.

All patient records were collected and contained the following data: anamnesis, state of the patient according to an objective physical examination, measurements of respiratory and heart rate, arterial blood pressure, a 12-canal EKG was obtained for all patients, and peripheral pulse oximetry (SpO_2_).

After the initial measurements of these values, in accordance with the decision made by the attending physician, the patients were treated with standard protocol therapy for acute cardiogenic pulmonary edema. According to the existing protocol, this includes the following: high-flow oxygen administered by a face mask (100% oxygen, 8–12 L/min), intravenous diuretics (furosemide), sublingual nitroglycerin, intravenous morphine if required, and the placement of a urinary catheter in order to monitor dialysis. Following standard therapy protocol, if the patient still met the criteria, noninvasive CPAP therapy was applied with positive end-expiratory pressure (PEEP) and the fraction of inhaled oxygen (FiO_2_) was set. With the CPAP mask set and working, patient transport to a secondary or tertiary care facility was started. During transport, adverse effects, the termination of CPAP therapy, or the need for endotracheal intubation were monitored. The duration of transport and CPAP application were also noted. All vital signs were measured at the end of transport, right before patient handover: the state of the patient according to an objective physical examination, measurements of respiratory and heart rate, arterial blood pressure, a 12-canal EKG was obtained for all patients, and peripheral pulse oximetry (SpO_2_). Changes in SpO_2_ and respiratory rate were recorded at three points: initial, after standard therapy, and after CPAP administration. CPAP was continued until hospital arrival if no criteria for the termination of CPAP were fulfilled.

The primary outcomes of the study focused on clinical improvement in patients with acute cardiogenic pulmonary edema treated with CPAP during prehospital management. These included the resolution of respiratory distress, evidenced by a decrease in respiratory rate and an improvement in dyspnea severity scores; a reduction in pCO_2_ levels, indicating improved ventilation; and the successful avoidance of endotracheal intubation in all patients. Additionally, oxygenation improved significantly, as reflected by increased SpO_2_ levels, and hemodynamic stabilization was achieved through the management of blood pressure and heart rate.

### 2.3. Statistical Analysis

When data were distributed normally, the mean value and the standard deviation are depicted; if the data were not distributed normally, the median as well as the interquartile range are depicted. Comparisons between continuous variables were made using the *t*-test (normally distributed data), the Wilcoxon test (non-normally distributed data), as well as ANOVA. Differences between categorical data were compared by means of a Chi-square test. The significance level was set at 0.05.

## 3. Results

A total of 50 patients with acute cardiogenic pulmonary edema treated by physician-led emergency medical teams in the Canton of Sarajevo were included in the study. Of these, 21 (42.0%) were male, and 29 (58.0%) were female. The median age of the patients was 74.0 (69.0;84.0) years, ranging from 50 to 90 years old. Among the existing comorbidities in these patients, hypertension was present in 33 patients (66.0%), diabetes mellitus in 16 (32.0%), chronic obstructive pulmonary disease (COPD) in 14 (28.0%), heart failure in 18 (36.0%), and dyslipidemia in 12 (24.0%). Additionally, two patients (4.0%) had a history of previous pulmonary thromboembolism, two (4.0%) had renal insufficiency, and two (4.0%) had experienced a previous ischemic cerebrovascular event. All data regarding demographic and clinical characteristics of patients are presented in Table 1.

### Clinical Parameters of Patients with Acute Cardiogenic Pulmonary Edema Treated with CPAP During Prehospital Management by Physician-Led Emergency Teams in Sarajevo Canton

Upon initial contact, mean systolic blood pressure significantly decreased between initial contact and before transportation (151.0 ± 41.0 vs. 138.4 ± 32.0, *p* < 0.001), and between the period prior to transportation and before hospital admission (138.4 ± 32.0 vs. 130.2 ± 28.5, *p* < 0.001).

Diastolic blood pressure followed a similar trend, declining from 85.6 ± 17.2 mmHg at initial contact to 81.1 ± 15.2 mmHg before transportation (*p* < 0.018), and to 80.2 ± 13.9 mmHg upon hospital admission (*p* = 0.083), after CPAP was instituted.

Heart rate also showed a downward trend, starting at 114 ± 26.4 beats per minute (bpm) and decreasing to 111.3 ± 24.9 bpm prior to transportation (*p* = 0.003), reaching 99.5 ± 18.2 bpm before hospital admission (*p* < 0.001), after CPAP was instituted.

The respiratory rate exhibited a notable decrease, from 31.0 ± 10.2 breaths per minute at initial assessment to 28.0 ± 10.5 bpm before transportation (*p* = 0.002) and 22.6 ± 7.3 bpm before hospital admission (*p* < 0.001), after CPAP was instituted.

End-tidal carbon dioxide (ETCO_2_) levels, on the other hand, showed an upward trajectory, rising from 28.0 mmHg (23.5; 33.5) on initial contact to 30.0 mmHg (27.0; 34.7) prior to transportation, and further to 35.0 mmHg (32.0; 37.5) before hospital admission. The results revealed a significant increase in ETCO_2_ between initial contact and before transportation (z = −4.144, *p* < 0.001) and between the period before transportation and before hospital admission (z = −3.782, *p* < 0.001), after CPAP was instituted.

Oxygen saturation (SpO_2_) improved notably during prehospital treatment, increasing from 79.0% (72.0; 81.0) on initial contact to 84.0% (80.0; 89.0) before transportation and reaching 94.0% (91.0; 98.2) prior to hospital admission. The results revealed a significant increase in SpO_2_ between initial contact and before transportation (z = −5.096, *p* < 0.001) and between the period prior to transportation and before hospital admission (z = −5.235, *p* < 0.001).

Electrocardiographic findings remained consistent throughout the prehospital management. Sinus rhythm was documented in 26 patients (52.0%) at all time points, while atrial fibrillation persisted in 23 patients (46.0%). Pacemaker rhythm was present in 1 patient (2.0%), and signs of myocardial ischemia were observed in 10 patients (20.0%) across all assessments.

The visual analog scale (VAS) score for dyspnea significantly decreased over time, from 8.0 (6.0; 8.2) at initial contact to 6.0 (4.0; 8.0) prior to transportation, and to 4.0 (3.0; 5.0) before hospital admission. The results revealed a significant decrease in VAS score for dyspnea between initial contact and before transportation (z = −4.663, *p* < 0.001) and between period before transportation and before hospital admission (z = −5.039, *p* < 0.001).

All data regarding changes in the clinical parameters of patients with cardiogenic pulmonary edema treated with CPAP during prehospital management by physician-led emergency teams in the Canton of Sarajevo are presented in Table 2.

## 4. Discussion

To the best of our knowledge, this is the first study on prehospital CPAP utilization in Bosnia and Herzegovina. For the first time in the Canton of Sarajevo, 50 patients with acute cardiogenic pulmonary edema were treated with noninvasive CPAP ventilation. Incorporating CPAP into standard therapy protocols was associated with favorable patient outcomes, including reductions in systolic and diastolic blood pressure, heart rate, and respiratory rate, alongside increases in end-tidal CO_2_ and peripheral oxygen saturation levels across all three measuring points. Patients also reported subjective improvements, including reduced dyspnea and an overall sense of better well-being. The study further highlighted the importance of early CPAP application, even during short transport times, demonstrating significant improvements in clinical parameters and overall patient condition in transport times of less than 20 min.

A study conducted by Hensel et al. showed similar results with patients divided into two time frame groups (NIV-group 1: ≤15 min, NIV-group 2: >15 min)—both showing the stabilizing effect of NIV in terms of vital parameters [11]. The data on the in-hospital use of CPAP for acute cardiogenic pulmonary edema are extensive; however, physician-guided CPAP therapy utilized in prehospital settings is scarcely described in the literature, most probably due to the fact that not many countries have an emergency medical service system which includes physicians in the field. Additionally, previous studies problematize the accuracy of acute cardiogenic pulmonary edema diagnosis in paramedic-operated EMS systems, finding that the diagnosis of cardiogenic pulmonary edema was corroborated by the ED or in-hospital physician in 13 patients (68%) [12,13]. Williams et al. reported that only 41% of patients, who were coded as suffering acute pulmonary edema by a paramedic had this particular emergency department discharge diagnosis [14]. Establishing a correct working diagnosis of acute cardiogenic pulmonary edema in the field, without the possibility of additional tests such as an X-ray, CT scan, or laboratory gas analysis, can be extremely challenging and therefore defining an indication for CPAP therapy can be an issue too. In our study, physicians in mobile unit ambulances of the EMS were the ones determining whether a patient was suffering from acute cardiogenic pulmonary edema, and the presumed diagnosis was later checked in the patient’s unified electronic record. We found that the final diagnosis for our patients matched the prehospital physician’s presumed diagnosis in 97% of cases. Previous studies of prehospital CPAP therapy use have not recorded any major adverse events. The adverse events of prehospital CPAP that have been described in the literature include: loss of consciousness, worsening dyspnea, apnea, hemodynamic instability, and making an initially missed or new pneumothorax worse [15,16]. In our study, hemodynamic instability occurred in one patient, manifesting as a systolic blood pressure under <80 mmHg, and a high-risk paroxysmal supraventricular tachycardia occurred in another. Another study also reported the discontinuation of CPAP therapy due to hypotension, nausea, and the worsening of dyspnea [17]. We consider it likely that the hypotension in one patient may have been due to an excessive dosage of intravenous furosemide, seeing as the diuresis in this patient exceeded 1 L in a very short transport time of 15 min. However, both cases were treated accordingly, and CPAP therapy was discontinued.

Peripheral oxygen saturation and respiratory rate were measured at three different time points—an initial value (initial contact), after the standard treatment protocol (before patient transport begins), and after CPAP administration (right before hospital admission). Please see Table 2.

Measured by the progress of peripheral oxygen saturation and respiratory rate, the efficacy of CPAP therapy is clearly visible. Several studies have shown that the addition of CPAP ventilation therapy to standard therapeutic protocol leads to improved peripheral oxygen values and respiratory rate [17,18,19,20]. The comparison of standard medical therapy plus CPAP to standard medical therapy alone has shown that CPAP is superior when using SpO_2_ or respiratory rate as measures of effectiveness in patients with acute cardiopulmonary edema or acute respiratory failure [21,22,23]. Finn et al. found that the respiratory rate was lowered in patients treated with CPAP for severe respiratory distress, and that levels of tachypnoea and dyspnea were also reduced [18]. However, two prehospital studies in patients with respiratory distress did not find any benefit of CPAP when comparing groups before and after implementation [22,23]. All three above-mentioned studies examined the use of CPAP in mixed causes of acute respiratory failure—mainly acute cardiogenic pulmonary edema and chronic obstructive pulmonary disease. All of the patients in our study received standard treatment protocol drugs prior to the application of CPAP ventilation. An important factor to rule in is the specific drugs administered to patients with acute cardiogenic pulmonary edema—diuretics (mainly intravenous furosemide), morphine, vasodilators (sublingual nitroglycerin) at fairly high administration rates, similarly to other physician-led European EMS systems [24,25,26]. On the other hand, in paramedic-led EMS systems, namely in the United States of America from where the majority of studies on the prehospital administration of CPAP originate, the administration of diuretics and vasodilators varies from almost none to 47.7% [25] for diuretics, and 27% [26] to 86.4% [27] for vasodilators. Morphine is administered significantly less frequently in the USA than in European EMS systems [28,29,30,31]. This may also account for the different results seen from system to system when it comes to the utilization of CPAP ventilation in prehospital settings.

This study was too small to be able to assess whether and how the use of prehospital CPAP would affect patient mortality. A systematic review published by Williams et al. found a reduction in the number of intubations and mortality in patients with acute respiratory failure who received CPAP in the prehospital setting [14]. Also, the authors emphasize that, due to inherent differences in the organization and staffing of EMS systems, the results they found may not be applicable to other healthcare systems. Due to this, we believe additional studies on the prehospital use of CPAP in physician-staffed emergency medical services would be warranted to assess its effects on the length of hospitalization and mortality. Additionally, exploring the effects of prehospital CPAP administration on these parameters in different etiologies of acute respiratory failure—such as chronic obstructive pulmonary disease exacerbations and asthma—could also contribute to the increase in CPAP therapy indications outside of hospital utilization.

The strength of this study includes a small margin of error when it comes to the presumed prehospital diagnosis of acute cardiogenic pulmonary edema in prehospital settings matching the final diagnosis of patients—we attribute this to the presence of physicians in the EMS system of the Canton of Sarajevo. This was a challenge from many other studies, seeing as how systems differ in terms of staffing [32,33]. Another limitation of the study is that patient inclusion was limited to ambulances with a CPAP machine in their equipment—seeing that as, at the time of the study, not all ambulances were equipped with it, causing a potential selection bias. Also, the physician in the ambulance car was the treatment provider and data collector—inducing observer bias. The data were gathered on a day-to-day basis, imposing no difference in the treatment of patients, simply adding an additional step to standard protocol. However, this resulted in a relatively low number of patients and warrants an additional, prospectively controlled study with a larger number of cases.

## 5. Conclusions

In a physician-led EMS system, prehospital CPAP treatment administered to patients with acute cardiogenic pulmonary edema in the field is highly effective and safe. Patients treated with CPAP as an addition to standard care protocol had a larger increase in peripheral pulse oximetry and a reduced respiratory rate even during short, urban setting prehospital transport to the hospital. This line of treatment can therefore be implemented as an additional stage in the standard treatment protocol. However, a larger scale study is needed in order to assess long-term effects and the impact on mortality of prehospital CPAP administration.

## Figures and Tables

**Table 1:** Demographic and clinical characteristics of patients presented with acute cardiogenic pulmonary edema.

Variable		N	%
**Sex**	Male	21	42.0
	Female	29	58.0
**Age (median, 25th, 75th percentile)**		74.0 (69.0; 84.0)	
**Comorbidities**		**N**	**%**
	Hypertension	33	66.0
	Diabetes mellitus	16	32.0
	Chronic obstructive pulmonary disease (COPD)	14	28.0
	Heart failure (HF)	18	36.0
	Dyslipidemia	12	24.0
	Previous PTE	2	4.0
	Renal insufficiency	2	4.0
	Previous ischemic cerebrovascular event (ICV)	2	4.0

**Table 2:** Changes in clinical parameters of patients with cardiogenic pulmonary edema treated with CPAP during prehospital management by physician-led emergency teams in Sarajevo Canton.

Variable		Initial Contact	Following Standard Treatment Protocol	Following CPAP Administration
**Blood pressure (mmHg)**	Systolic	151.0 ± 41.0	138.4 ± 32.0 *	130.2 ± 28.5 **
	Diastolic	85.6 ± 17.2	81.1 ± 15.2 *	80.2 ± 13.9
**Heart rate**		114 ± 26.4	111.3 ± 24.9 *	99.5 ± 18.2 **
**Respiratory rate (cycles/min)**		31.0 ± 10.2	28.0 ± 10.5 *	22.6 ± 7.3 **
**ETCO_2_ (mmHg)**		28.0 (23.5; 33.5)	30.0 (27.0; 34.7) *	35.0 (32.0; 37.5) **
**SPO_2_ (%)**		79.0 (72.0; 81.0)	84.0 (80.0; 89.0) *	94.0 (91.0; 98.2) **
**ECG (N,%)**	Sinus rhythm	26 (52.0)	26 (52.0)	26 (52.0)
	Atrial fibrillation	23 (46.0)	23 (46.0)	23 (46.0)
	Pacemaker rhythm	1 (2.0)	1 (2.0)	1 (2.0)
	Ischemia signs	10 (20.0)	10 (20.0)	10 (20.0)
**VAS score**		8.0 (6.0; 8.2)	6.0 (4.0; 8.0) *	4.0 (3.0; 5.0) **

* Significant difference between initial contact and before transportation. ** Significant difference between before transportation and before hospital admission.

## Data Availability

Data available upon request.
